# Comparison of treatment recompression tables for neurologic decompression illness in swine model

**DOI:** 10.1371/journal.pone.0266236

**Published:** 2022-10-05

**Authors:** W. Rainey Johnson, Nicholas G. Roney, Hanbing Zhou, Geoffrey E. Ciarlone, Brian T. Williams, William T. Green, Richard T. Mahon, Hugh M. Dainer, Brett B. Hart, Aaron A. Hall

**Affiliations:** 1 Naval Medical Research Center, Bethesda, Maryland, United States of America; 2 Henry M. Jackson Foundation, Bethesda, Maryland, United States of America; University of North Carolina at Chapel Hill, UNITED STATES

## Abstract

**Background:**

Significant reductions in ambient pressure subject an individual to risk of decompression illness (DCI); with incidence up to 35 per 10,000 dives. In severe cases, the central nervous system is often compromised (>80%), making DCI among the most morbid of diving related injuries. While hyperbaric specialists suggest initiating recompression therapy with either a Treatment Table 6 (TT6) or 6A (TT6A), the optimal initial recompression treatment for severe DCI is unknown.

**Methods:**

Swine were exposed to an insult dive breathing air at 7.06 ATA (715.35 kPa) for 24 min followed by rapid decompression at a rate of 1.82 ATA/min (184.41 kPa/min). Swine that developed neurologic DCI within 1 hour of surfacing were block randomized to one of four United States Navy Treatment Tables (USN TT): TT6, TT6A-air (21% oxygen, 79% nitrogen), TT6A-nitrox (50% oxygen, 50% nitrogen), and TT6A-heliox (50% oxygen, 50% helium). The primary outcome was the mean number of spinal cord lesions, which was analyzed following cord harvest 24 hours after successful recompression treatment. Secondary outcomes included spinal cord lesion incidence and gross neurologic outcomes based on a pre- and post- modified Tarlov assessment. We compared outcomes among these four groups and between the two treatment profiles (i.e. TT6 and TT6A).

**Results:**

One-hundred and forty-one swine underwent the insult dive, with 61 swine meeting inclusion criteria (43%). We found no differences in baseline characteristics among the groups. We found no significant differences in functional neurologic outcomes (p = 0.77 and 0.33), spinal cord lesion incidence (p = 0.09 and 0.07), or spinal cord lesion area (p = 0.51 and 0.17) among the four treatment groups or between the two treatment profiles, respectively. While the trends were not statistically significant, animals treated with TT6 had the lowest rates of functional deficits and the fewest spinal cord lesions. Moreover, across all animals, functional neurologic deficit had strong correlation with lesion area pathology (Logistic Regression, p < 0.01, Somers’ D = 0.74).

**Conclusions:**

TT6 performed as well as the other treatment tables and is the least resource intensive. TT6 is the most appropriate initial treatment for neurologic DCI in swine, among the tables that we compared.

## Introduction

Substantial reductions in ambient pressure put an individual at risk for decompression illness (DCI). DCI can be divided into decompression sickness (DCS) and arterial gas embolism (AGE). While difficult to distinguish during acute presentation, pathophysiology of DCS and AGE is different. DCS is thought to arise when previously dissolved gases (mostly inert gas) in tissue reaches a state of supersaturation created during exposure to rapid decreases in ambient pressure. This supersaturation state is necessary for bubble formation. Once a nidus of bubbles is formed, a host of poorly characterized downstream reactions occur that manifest as DCS symptoms [1]. In contrast, AGE generally arises from pulmonary over-inflation syndromes, where pressure differentials as little as 0.1 atmospheres absolute (ATA), equivalent to 10.13 kilopascals (kPa), between the intratracheal and intrapleural spaces can exist, leading to barotrauma and alveolar rupture [1]. If the inhaled, pressurized gas enters ruptured blood vessels, return to the heart and enter systemic circulation can form an arterial gas embolus, potentially blocking arterial blood flow and leading to ischemia [1]. The most severe forms of DCS and AGE present similarly and may present acutely with life-threatening impairments, affecting the nervous, respiratory, or circulatory systems.

Severe DCI impairments impact the nervous and/or cardiopulmonary systems with a wide range of presentations, including headache, vertigo, blindness, nystagmus, numbness, paresthesia, paralysis, dyspnea, seizures, and, at the extreme, cardiovascular collapse and death [1,2]. Among those organ systems afflicted by severe DCI, the central nervous system (CNS) is compromised in the vast majority of cases (>80%), making CNS injury among the most morbid of diving related injuries. In examination of the subsets of DCI, neurologic DCS and AGE incidents most often affect the spinal cord (~70%) and brain, respectively [1–4]. The gold standard treatment for DCI is hyperbaric oxygen therapy which is a subset of recompression therapy.

**Fig 1:**
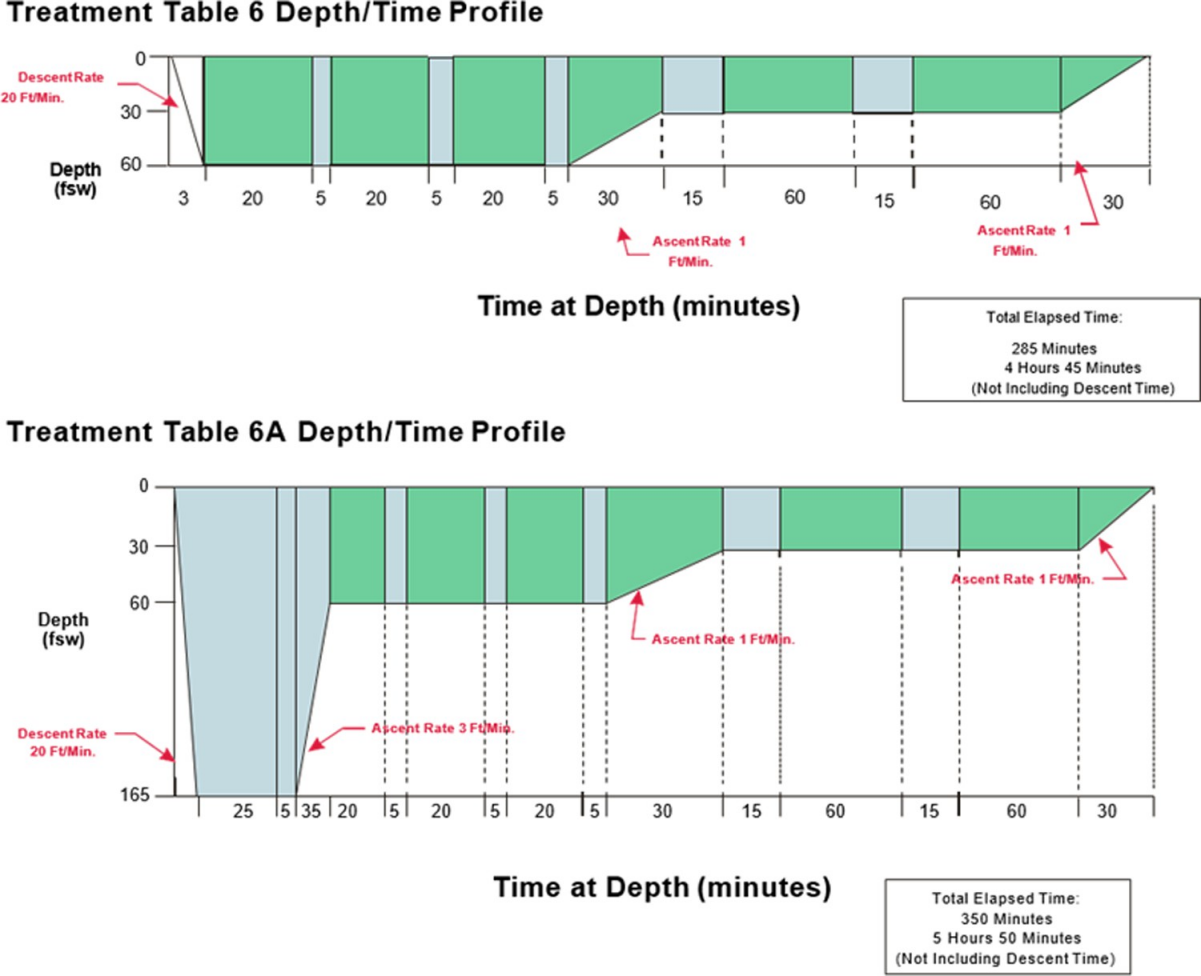
Adapted from US Navy Diving Manual, Revision 7. Depicts dive profile for USN TT6 (A) and USN TT6A (B).

In general, recompression therapy serves to increase ambient pressure (to physically decrease bubble size) and, based on the treatment gas, may augment inert gas elimination. Since the inception of recompression therapy, various schedules of increased pressure and time have been devised, mostly empirically [5,6]. Animal models have also played a significant role in the development and comparisons recompression treatments [5,7,8]. The United States Navy (USN) developed recompression treatment tables in the 1960s and published them in the first version of the US Navy Diving Manual in 1975 [6,9]. The most recent USN Diving Manual recommends both the USN Treatment Table 6 (TT6) or USN Treatment Table 6A (TT6A), shown in **Fig 1A & 1B**, as treatments for severe (e.g. neurologic) DCS and AGE depending on the patient’s initial response to a TT6 [3]. While hyperbaric specialists usually initiate treatment with either a TT6 or TT6A based on empiric preference or facility convention (local standard of care), optimal initial recompression treatment for severe DCI is contested.

Theoretically, breathing oxygen at 2.82 ATA (285.74 kPa) in a TT6 recompression at a depth of 60 feet of sea water (fsw), or 2.82 ATA (285.74 kPa), balances the benefits of inert gas elimination against oxygen toxicity (seizures and pulmonary compromise). While 165 fsw initial depth (6 ATA, 607.95 kPa) in a TT6A recompression offers greater bubble reduction by decreasing its diameter by 45% and volume by 83%, diffusion gradients driving inert gas elimination are less than or similar to a TT6 depending on the treatment gas used (e.g. air v. heliox or nitrox) [1]. Despite empirical support and strong advocates for both strategies, TT6 and TT6A have never been compared head-to-head in a prospective, randomized controlled trial for the treatment of neurologic DCI [10–12].

## Methods

### Ethics statement

All experiments were conducted according to the principles set forth in the “Guide for the Care and Use of Laboratory Animals,” Institute of Laboratory Animal Resources, National Research Council, National Academy Press, 1996. The study protocol (17-OUMD-14L) was reviewed and approved by the Walter Reed Army Institute of Research/Naval Medical Research Center Institutional Animal Care and Use Committee in compliance with all Federal regulations governing the protection of animals and research. The health status of animals was monitored daily, and the research was conducted in a facility accredited by the Association for Assessment and Accreditation of Laboratory Animal Care-International. Euthanasia was carried out in accordance with the recommendations and guidelines of the American Veterinary Medical Association.

### Animal selection and facility acclimatization

Castrated Yorkshire swine were procured from a single vendor for all experiments (Animal Biotech Industries, Danboro, PA) and acclimatized for at least 5 days prior to any experiment. Animals were housed in a 3.5 x 10ft run with free access to water and food (“Lab Diet”, from PMI Nutrition LLC, Brentwood, MO) in addition to various forms of environmental enrichment. Lighting was maintained using a daily 12:12hr on/off light schedule.

### Experimental approach

Despite the severe outcomes of neurological injury from DCI, human treatment trials are limited by issues of wide geographical distribution, sporadic occurrence and blinding to randomization. Instead of relying on limited human data, we used a previously characterized swine model of neurological DCI. We sought to directly address the knowledge gap by comparing neurologic DCI outcomes in a randomized controlled trial of recompression treatment with TT6, TT6A-air (21% oxygen, 79% nitrogen), TT6A-nitrox (50% oxygen, 50% nitrogen), and TT6A-heliox (50% helium, 50% oxygen) with this proven swine model of neurological DCI [4]. TT6 and TT6A-air are standard treatments borrowed widely from the US Navy Diving Manual, which also discusses the use of nitrox and heliox gases for some diving applications. The 50:50 mixtures described in the manual were selected to keep the maximum partial pressures of oxygen near equivalent across TT6, TT6A-nitrox, and TT6A-heliox, while simultaneously limiting risk of central nervous system oxygen toxicity [3,13]. We utilized a superiority study design and tested the hypothesis that there would be a difference in treatment outcomes among the treatment groups–TT6, TT6A-air, TT6A-nitrox, and TT6A-heliox–and a difference between treatment profiles–TT6 and TT6A (i.e. TT6A-air, TT6A-nitrox, and TT6A-heliox). Furthermore, we hypothesized, based on the theoretical strengths and weaknesses of each treatment table and treatment gases, that TT6 and TT6A-heliox tables would be the most effective treatments for neurologic DCI following a provocative air dive.

### Animal preparation

On the day of experiment, the swine walked on a treadmill and a modified Tarlov score was given (13,14). Each swine performed at least one modified Tarlov assessment prior to his insult dive. For the modified Tarlov assessment, swine were placed on the treadmill and the speed gradually increased to 1 mph, while their walking quality was observed for up to 5 minutes. Recorded Tarlov scores ranged from 0 to 6, where 0 represented complete hindlimb paralysis, 5 normal walking at 1 mph, and 6 normal walking at 1 mph sustained for 5 minutes [14,15]. Scores of 6 were considered normal. The researchers repeated this baseline assessment only when an animal demonstrated difficulty with treadmill acclimatization, such as resisting walking, and did not achieve a score of 6; all swine a score of 6 prior to diving.

Subsequently, each animal had an ear vein catheter placed (20–22 G, Becton, Dickson, and Company, Franklin Lakes, NJ) weight recorded, with baseline heart rate (ECG, ADI Power Lab and Bio Amplifier, Colorado Springs, CO), pulse oxygen saturation (BCI 3401 Fingerprint Pulse Oximeter, Smiths Medical, Dublin, OH), respiratory rate, and temperature monitored for 5 minutes. A detailed neurologic exam was performed with focus on strength, reflexes, seizure activity, and nystagmus. Next, a custom fitted jacket was applied to the experimental animal and the swine placed inside a custom wire cage, which was then placed inside a hyperbaric chamber (floodable volume 45 cu ft) to undergo the insult dive.

### Insult dive

Swine (n = 1/dive) were pressurized according to the following insult dive profile: 0.91 ATA/min (92.21 kPa/min) descent, 24 min at 7.06 ATA (715.35 kPa), and 1.82 ATA/min (184.41 kPa/min) ascent. Throughout the insult dive, animals breathed chamber air. The dive profile was selected based on a standardized, previously developed swine model for neurologic DCI [4]. The swine were visually monitored for distress using a closed-circuit video recording system.

### DCI diagnosis

Immediately upon surfacing, swine were removed from the chamber and wire cage. They were then placed in a Panepinto sling (Panepinto & Associates, Loveland, CO). Oxygen was delivered (15 L/min, 100% oxygen) via a nose cone mask (Jorgensen Labs, Loveland, CO). Heart rate, respiratory rate, and temperature were recorded at five-minute intervals. Investigators monitored each swine for signs and symptoms of neurologic DCI, which included assessing for nystagmus, cutis marmorata, and seizure activity, as well as testing for weakness, reflexes, and paralysis both in and out of the sling. Major criteria included paralysis, seizure, and nystagmus that started after surfacing. Minor criteria included cutis marmorata, weakness, or loss of reflexes. A diagnosis of neurologic DCI was given with the presence of one major finding or two minor findings (**Table 1**). Swine were observed for onset of neurologic DCI for a maximum of one hour.

**Table 1:** Shows the diagnostic criteria used to diagnose neurologic DCI.

**Major Criteria**	**Diagnosis of Neurologic DCI** 1 Major Criteria **OR** 2 Minor Criteria
Seizure Nystagmus Paralysis	
**Minor Criteria**	
Weakness Areflexia Cutis Marmorata	

Animals manifesting signs and symptoms of neurologic DCI during the one-hour observation window received a single intravenous bolus of 8 mg diazepam (Medisca, Plattsburgh, NY) and 20 mg xylazine (Patterson Vet Supply, Inc, Greeley, CO), on surface while in the Panepinto sling prior to closing and pressurization of the hyperbaric chamber. The swine were then randomized to one of four treatment groups: TT6, TT6A-air, TT6A-nitrox, and TT6A-heliox. Block randomization was performed by chamber operators and blinded to investigators until the start of recompression therapy. To account for a standard surface interval prior to surface recompression treatment, recompression started at least 5 minutes after surfacing from the insult dive [3]. Swine that did not show signs and symptoms of neurologic DCI during the one-hour observation window were monitored for an additional 24 hours for late-onset neurologic DCI but were not enrolled in the study.

### Recompression treatment

Depending upon their randomized treatment table assignment, swine were recompressed according to the standard U.S. Navy time and depth profiles specified for the TT6 or TT6A (**Fig 1A & 1B**). The corresponding treatment gas (i.e. 100% oxygen, air, nitrox, or heliox) was delivered to the swine via secured nose cone. Sedation was maintained using a continuous diazepam (1–5 mg/hr) infusion with intermittent xylazine boluses (20 mg), with the latter given no more frequently than every 30 minutes. The investigators monitored chamber temperature and swine temperature, heart rate, and comfort during the recompression treatment. All swine that were diagnosed with Neuro-CI were assigned a recompression treatment table. Animals which did not complete the full recompression protocol were censored from subsequent data analysis.

### Post recompression assessment

After completing the assigned recompression treatment, intravenous sedation was discontinued and heart rate, pulse oxygen saturation, respiratory rate, and temperature were recorded at five-minute intervals. After 1 hour of observation, swine were returned to their run with free access to food and water and periodically assessed for neurologic status based on a modified Tarlov assessment and general well-being, including monitoring for skin sores, neurogenic bladder, and ability to access food/water.

Twenty hours after recompression therapy, treadmill-based neurologic assessments using a modified Tarlov scoring system were repeated. Swine were considered to have no neurologic deficit if with a score of 5 or 6 on the assessment. After 24 hours of post-treatment observation, the enrolled swine received 10,000 units heparin (Patterson Vet Supply, Inc, Greeley, CO) via ear vein and were humanely euthanized with ear vein injection (20–22 G, Becton, Dickson, and Company, Franklin Lakes, NJ) with Euthasol® (Patterson Vet Supply, Inc, Greeley, CO). After euthanasia, a board-certified veterinary pathologist performed a necropsy on each enrolled swine. The pathologist removed each spinal cord and sectioned it into nine segments of equal length. Pathology prepared two slides from each of these nine sections stained with hematoxylin and eosin. The slides were numbered 1–18 from cranial to caudal.

### Image acquisition & analysis

Images of the pathology slides were captured at 2.5x using ZEN 2 Pro (Blue Edition) through a Zeiss Axio Imager.M2 (Carl Zeiss Microscopy, LLC, Thornwood, NY). The white balance was manually set through the ZEN 2 Pro software with the white space surrounding the tissue sample used as the reference standard. Thereafter, automatic focusing was used. The raw data files were saved, converted to TIFF files, and exported. One investigator (WG) analyzed TIFF images of each slide via ImageJ (Rasband, W.S., ImageJ, U. S. National Institutes of Health, Bethesda, Maryland, USA, http://imagej.nih.gov/ij/, 1997–2011) for lesion area in collaboration with a board-certified veterinary pathologist. Using the polygon selection tool, WG cropped each image to include only the white matter, the relevant area of neurologic DCI pathology [4]. The remaining image was converted to an 8-bit image and the color threshold was adjusted such that the only particles selected were those resultants from the characteristic white marks of a lesion. The particles were then quantified through ImageJ and the area was divided by the total area of the white matter and expressed as the percent lesion area for each of the 18 spinal cord sections. The mean percent spinal cord lesion area for each animal was defined as the average of the percent lesion area for each section. The results were recorded in a spreadsheet (Excel, Microsoft) for downstream statistical analysis.

### Statistical analysis

We conducted a conservative power analysis, finding that 21 swine were needed to detect a 95% change in lesion area using one-way ANOVA with an alpha level of 0.025 and a power of 90% based on a previous study [14]. With an expected enrollment of 59%, 143 swine were expected to undergo the insult dive. The primary endpoint for this study was mean percent spinal cord lesion area compared among the treatment groups (i.e. TT6, TT6A-air, TT6A-nitrox, and TT6A-heliox) using one-way ANOVA and between treatment table profiles (i.e. TT6 v. TT6A) using t-test. A second analysis of the percent lesion area using each section as a data point was conducted between groups using one-way ANOVA with post-hoc Tukey testing where each spinal cord section was utilized as a data point. Secondary endpoints included Tarlov scores and spinal cord lesion incidence, which were analyzed using chi-square analysis with significance threshold of p<0.05. Baseline characteristics data was first assessed for normality using D’Agostino & Pearson testing. Parametric analyses were performed with one-way ANOVA or t- test with significance threshold of p<0.05. Non-parametric analysis was performed using Kruskal-Wallis and Mann-Whitney testing with a significance threshold of p<0.05. Time to diagnosis of neurologic DCI was analyzed using log-rank analysis. Correlation between post-dive Tarlov score and Lesion area was conducted using logistic regression where a Tarlov score of 6 was considered no Neurological Deficit and a Tarlov score of 5 or less was considered Neurological deficit. Data were analyzed using SAS ver9.4 (SAS, North Carolina) and GraphPad Prism ver 8.3.1. All statistics were completed by a statistician blinded to the treatment groups.

## Results

### Enrollment

**Fig 2:**
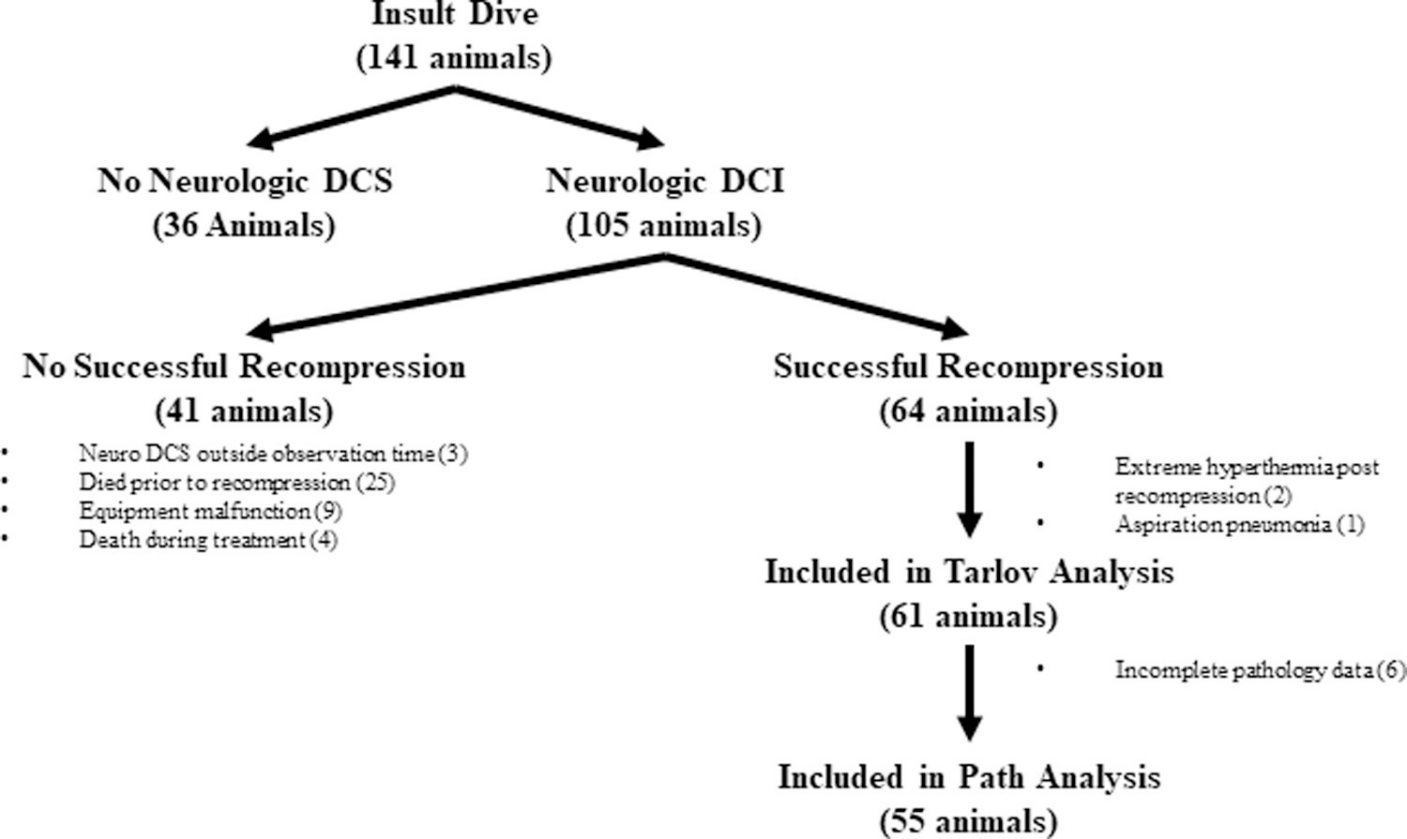
A flow diagram showing the animals included in the final statistical analyses and the reasons for animal exclusion.

One-hundred and forty-one swine underwent the insult dive, with 105 (74%) of these animals developing neurologic DCI and subsequently enrolled on the study. Of the enrolled subjects, 50 swine were censored from subsequent analysis. Due to rapid progression of neurologic DCI to cardiopulmonary DCS symptoms, twenty-five (24%) expired prior to undergoing their assigned recompression chamber treatment. Additionally, sixteen swine were excluded from the recompressed study group due to the following: equipment malfunction (e.g., broken breathing mask, n = 9), death during recompression treatment presumably from severe cardiopulmonary DCS (n = 4; 1 TT6, 2 TT6A-Air, 1 TT6A-Heliox) or late onset (i.e., great than the one hour observation window post-insult dive) of neurologic DCI symptoms (e.g., paralysis), such that animals did not immediately enter recompression therapy (n = 3) (**Table 2)**. Of the 64 animals that successfully completed recompression treatment, three animals expired shortly after treatment due to extreme hyperthermia (**Table 2**). Six of the remaining 61 animals had inadequate spinal cord pathology samples, leaving 55 for inclusion in the study histopathological analysis **(Fig 2)**. The analyses of initial neurologic DCI presentation, group characteristics, and gross neurologic functioning were similar between the 61 animals that successfully completed the protocol and the subset of 55 animals that had adequate pathology samples.

**Table 2:** Describes the rational for exclusion of animals that had neurologic DCI.

Reason for Exclusion	Number of animals	Amount of time spent in recompression treatment table	Comments and rationale
**Died prior to recompression**	25	None to Less than 3 minutes	These animals all had severe cardiopulmonary DCS. Even those that showed signs of neurologic DCS prior to their cardiopulmonary DCS died prior to reaching bottom. The outcome of these animals does not reflect the efficacy of the treatment tables. Therefore, the animals were excluded.
**Late onset neurologic DCS**	3	None	These animals had neurologic DCS manifesting as paralysis. Unfortunately, it occurred greater than 1 hour after the continuous observation period. The neurologic symptoms were identified during periodic observations and, therefore, these animals never had any recompression treatment and were excluded.
**Death during treatment**	4	3–317 minutes	These animals died during recompression treatment–TT6 (n = 1), TT6A-Air (n = 2), and TT6A-Heliox (n = 1). Three of the four occurred less than halfway into the recompression treatment. The one that occurred greater than halfway into recompression treatment had a full necropsy without clear etiology of death. Observation suggests that all of these were due to respiratory failure, possible from cardiopulmonary DCS. We opted for a per protocol analysis, rather than an intention-to-treat analysis, and therefore excluded these animals.
**Equipment malfunction**	9	3–330 minutes	All the equipment malfunctions resulted in the animals not receiving the appropriate treatment gas. Most (8) were due to a mask malfunction, such as disconnected hosing, valve break, or nose cone break. The last was due to a problem with gas flow that could not be troubleshooted sufficiently quickly, so the dive was aborted.
**Extreme Hyperthermia**	2	Completed	These animals had temperatures greater than 108°F after completing recompression therapy–TT6 (1) and TT6A-Air (1). They both occurred during the winter months, a few weeks apart. Despite genetic testing, no predisposition for malignant hyperthermia was found. No clear etiology identified. Excluded because unable to complete post-TT Tarlov assessment and presumption that the animals had unidentified underlying disorder (e.g., genetic, infectious, etc.).
**Aspiration pneumonia**	1	Completed	The animal completed the recompression treatment–TT6A-Air. However, upon surfacing, it was clear that the animal was in distress. Decision was made to humanely euthanize. Full necropsy showed large pulmonary consolidation, consistent with pneumonia. The animal was excluded from analysis because of poor underlying health.

### Pre-recompression treatment survivor characteristics

**Fig 3:**
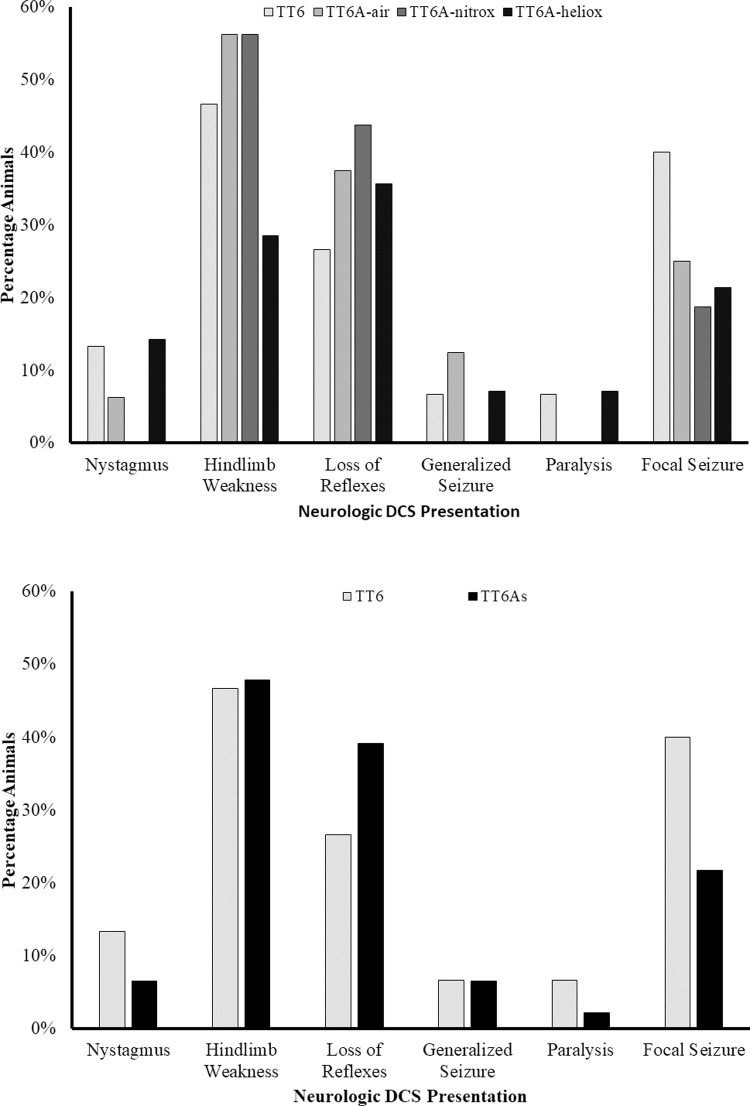
Bar graph showing the percentage of animals presenting with various signs and symptoms of neurologic decompression illness for each treatment group (A, Chi-square test, p = 0.81) and each treatment profile (B, Chi-square test, p = 0.69).

**Fig 4:**
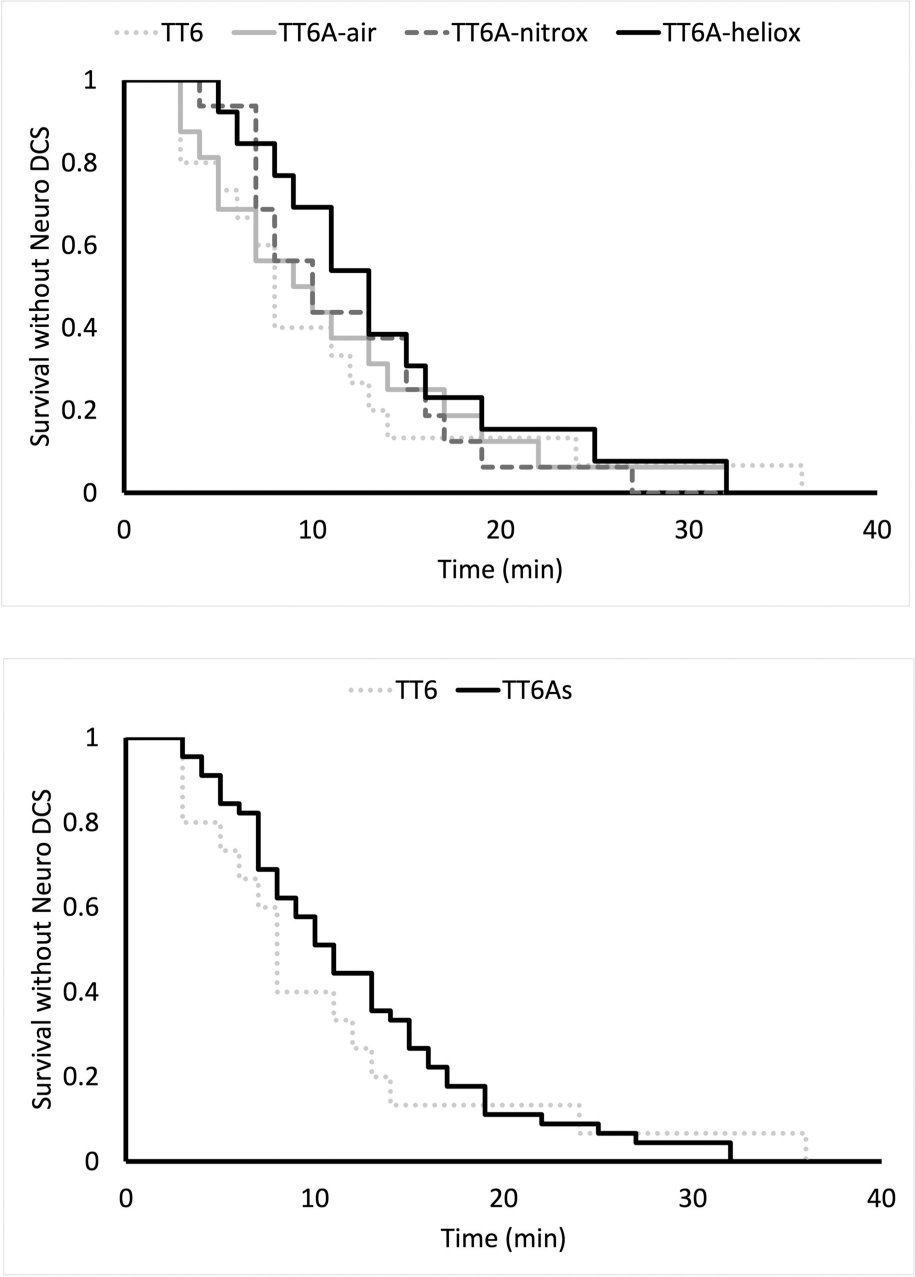
A Kaplan-Meier curve showing the latency to diagnosis of neurologic DCI in each treatment group (A, log rank, p = 0.83) and each treatment profile (B, log rank, p = 0.58).

Swine surviving to recompression included 15 animals that were subsequently treated with TT6, 16 with a TT6A-air, 16 with a TT6A-nitrox, and 14 treated with TT6A-heliox **(Table 3)**. Weight and Heart Rate but not SpO2 and Respiratory rate data passed normality test when evaluated using D’Agostino & Pearson test. One-way ANOVA analysis revealed no significant pre-recompression differences among groups with respect to weight (p = 0.35), heart rate (p = 0.60). Similarly, Kruskal-Wallis testing revealed no significant differences in respiratory rate (p = 0.19), or pulse oxygen saturation (p = 0.37) (**Table 3**). Similarly, a t-test demonstrated no differences in weight (p = 0.61) or heart rate (p = 0.28), while Mann-Whitney testing revealed no changes in respiratory rate (p = 0.07), or pulse oxygenation (p = 0.42) between treatment profiles (**Table 3**). The presenting symptoms of neurologic DCI did not differ among the groups or between treatment profiles respectively (Fisher’s exact test, p = 0.81 & p = 0.57, **Fig 3A & 3B**). The latency to diagnosis of DCI did not differ among groups or between treatment profiles respectively (Log-Rank, p = 0.83 & p = 0.57) **(Fig 4A & 4B).** There were no differences in baseline Tarlov scores among treatment groups or between treatment profiles, all animals received a maximum score.

**Table 3:** The baseline characteristics of the animals in each of the four treatment groups with no differences in baseline characteristics among treatment groups (ANOVA, p > 0.025, Kruskal Wallis, p>0.025) or between treatment profiles (t-test, p > 0.05, Mann-Whitney, p > 0.05).

Baseline Characteristics	Treatment Group								ANOVA, p-value among groups	Kruskal-Wallis, p-value among groups	t-test, p-value between profiles	Mann-Whitney, p-value between profiles
	TT6		TT6A-air		TT6A-nitrox		TT6A-heliox					
	*Mean*	*SD*	*Mean*	*SD*	*Mean*	*SD*	*Mean*	*SD*				
**Weight (kg)**	28.9	2.6	28.4	0.6	29.6	6.4	30.0	2.0	0.35		0.61	
**Heart Rate (bpm)**	135.3	26.6	125.9	0.3	128.2	18.7	132.3	19.3	0.60		0.28	
**Respiratory Rate (rpm)**	54.5	19.6	59.3	21.1	47.7	12.9	51.9	12.8		0.19		0.07
**SpO2 (%)**	96.5	4.2	95.7	7.3	96.6	3.4	96.7	2.7		0.37		0.42

### Post-recompression treatment clinical outcomes

**Fig 5:**
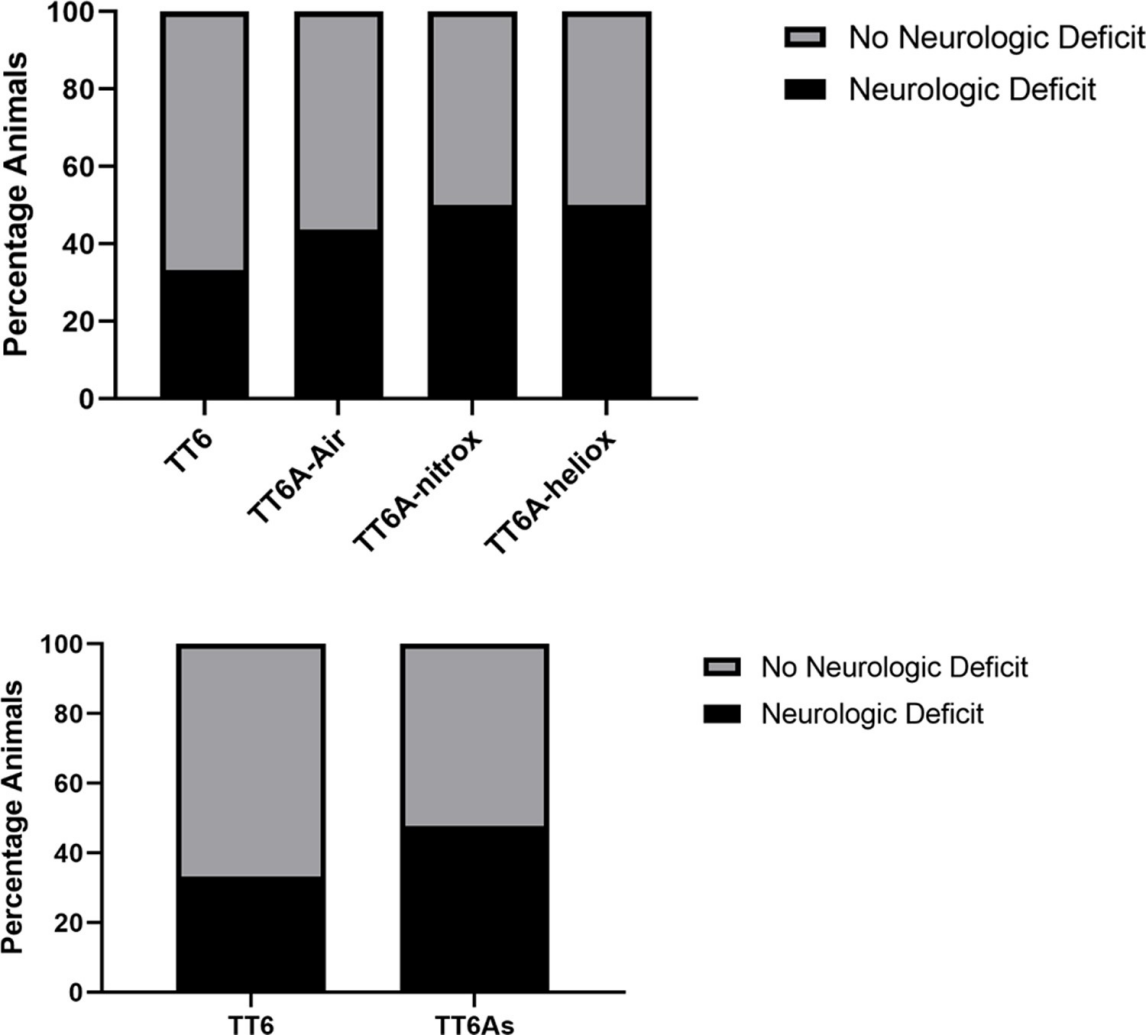
Bar graph showing the gross neurologic outcomes for animals in each treatment group (A, chi-square test, p = 0.77) and each treatment profile (B, chi-square test, p = 0.33, OR 1.83, 95% CI: 0.54–6.21) 24 hours post-insult and treatment.

There were no differences in post-treatment table heart rates, respiratory rates, or oxygen saturations among the four recompression groups (One-Way ANOVA, p = 0.29, Kruskal-Wallis, p = 0.19, and 0.37 respectively) or between the treatment profiles (t-test, p = 0.28, Mann-Whitney, p = 0.07, and 0.42). Fifty-six percent (n = 34) of animals had no gross neurologic deficit 24 hours post-insult dive and recompression treatment. However, the TT6 group had the largest percentage of animals without discernable deficit, with sixty-seven percent (n = 10) exhibiting no gross neurologic deficit after completing TT6 recompression treatment **(Fig 5A & 5B)**. Of those animals treated using a TT6A recompression profile, the percentages of animals without gross neurologic deficits were 56% (n = 9) after recompression treatment using TT6A-air and 50% after either TT6A-nitrox (n = 8) or TT6A-heliox (n = 7). Within treatment group comparison using chi-square testing revealed no significant differences in neurologic outcome (chi-square test, p = 0.77, **Fig 5A**). Similarly, there was no difference in gross neurologic outcomes between treatment profiles (chi-square test, p = 0.33, OR 1.83, 95% CI: 0.54–6.21, **Fig 5B**).

### Pathology–lesion area & incidence

The pathology data include 55 animals: 14 treated with TT6, 14 treated with TT6A-air, 15 treated with TT6A-nitrox, and 12 treated with TT6A-heliox. The pre-recompression treatment characteristics and presentations of neurologic DCI did not differ among the groups or between the profiles and were similar to the corresponding characteristics present for the 61 animals included in the overall neurologic outcome analyses. Specifically, the presenting symptoms of neurologic DCI in the 55 animals with complete pathology data did not differ among groups (chi-square test, p = 0.87, not graphically shown), nor did the latency to diagnosis of DCI (Log-rank, p = 0.67, not graphically shown). Similarly, one-way ANOVA analysis revealed no significant differences among groups for weight (p = 0.22), heart rate (p = 0.48), respiratory rate (p = 0.53), or pulse oxygen saturation (p = 0.99).

**Fig 6:**
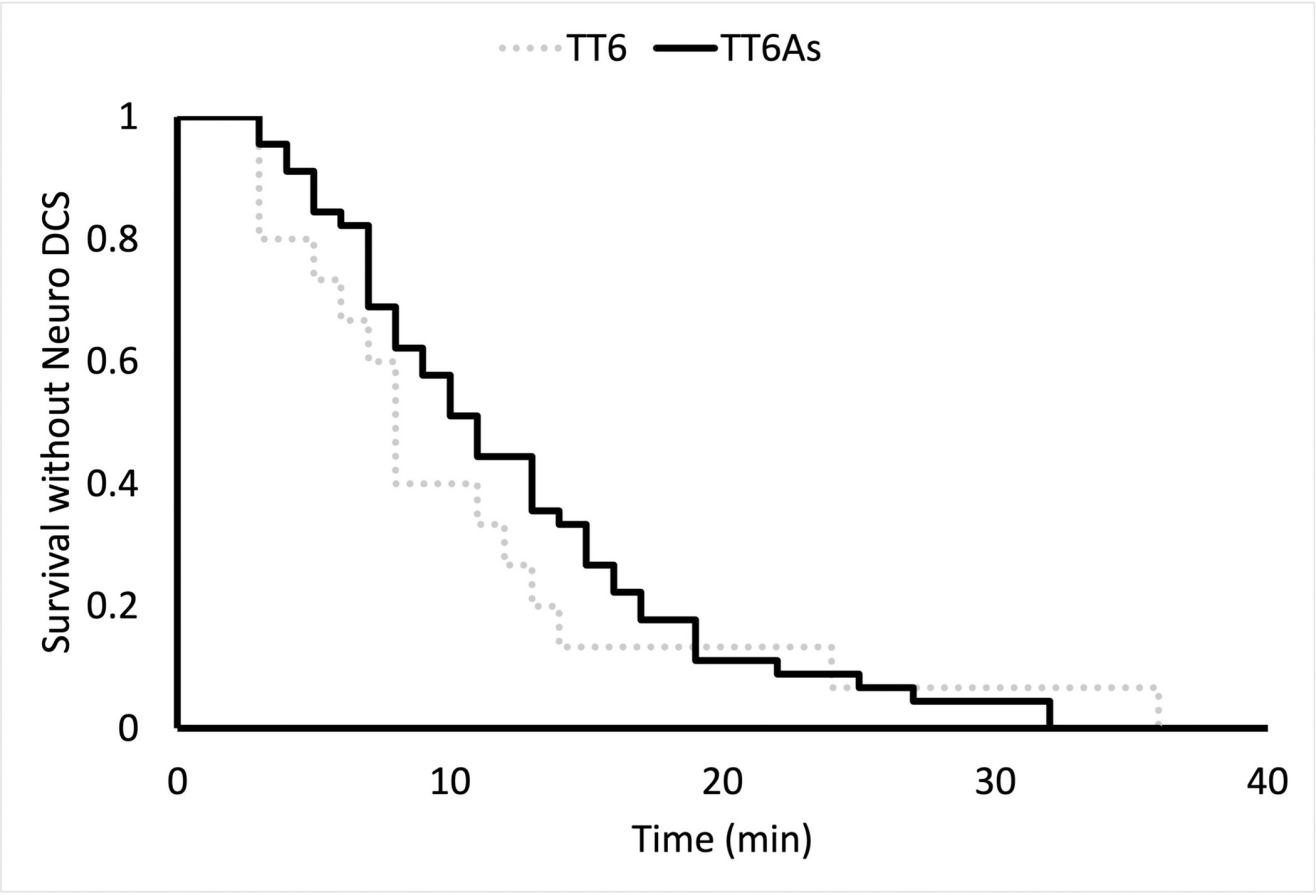
(A) Histogram showing incidence of spinal cord lesion across all animals included in the pathology analysis (55). (B) Histogram showing the mean spinal cord lesion area across all animals included in the pathology analysis.

**Fig 7:**
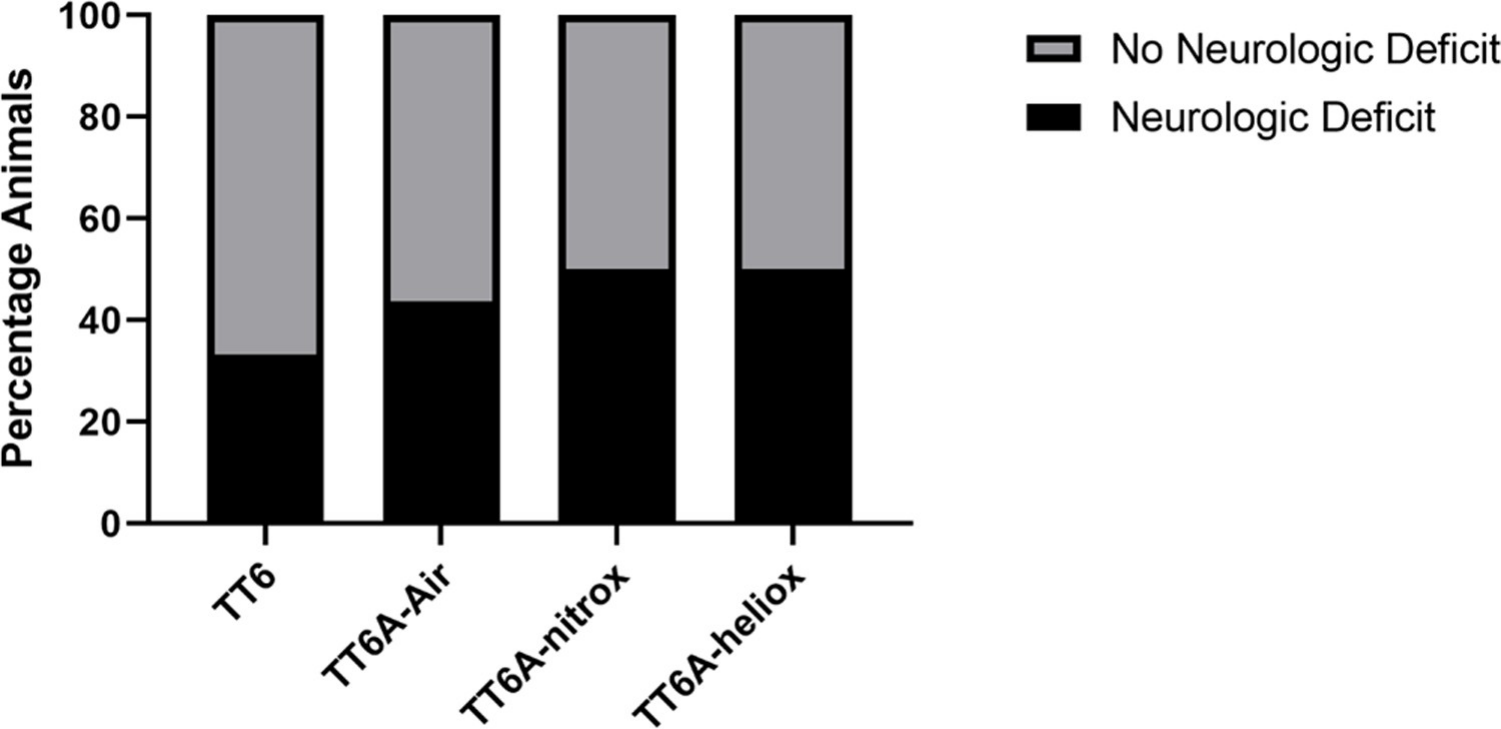
Shows spinal cord sectioning and representative pathologic samples for each spinal cord region and treatment table.

Qualitative comparison of the spinal cord histological samples identified the thoracic region of the spinal cord, roughly corresponding to slides numbers 7–12 **(Fig 6A)**, as the most severely damaged **(Fig 6B)**. Representative samples are shown in **Fig 7**. When the area and incidence of the lesions were quantified, the thoracic region had the greatest incidence and largest area of lesion across the 55 animals included in the pathology analysis. The distribution of the lesion incidence and mean areas was similar across all four groups.

**Fig 8:**
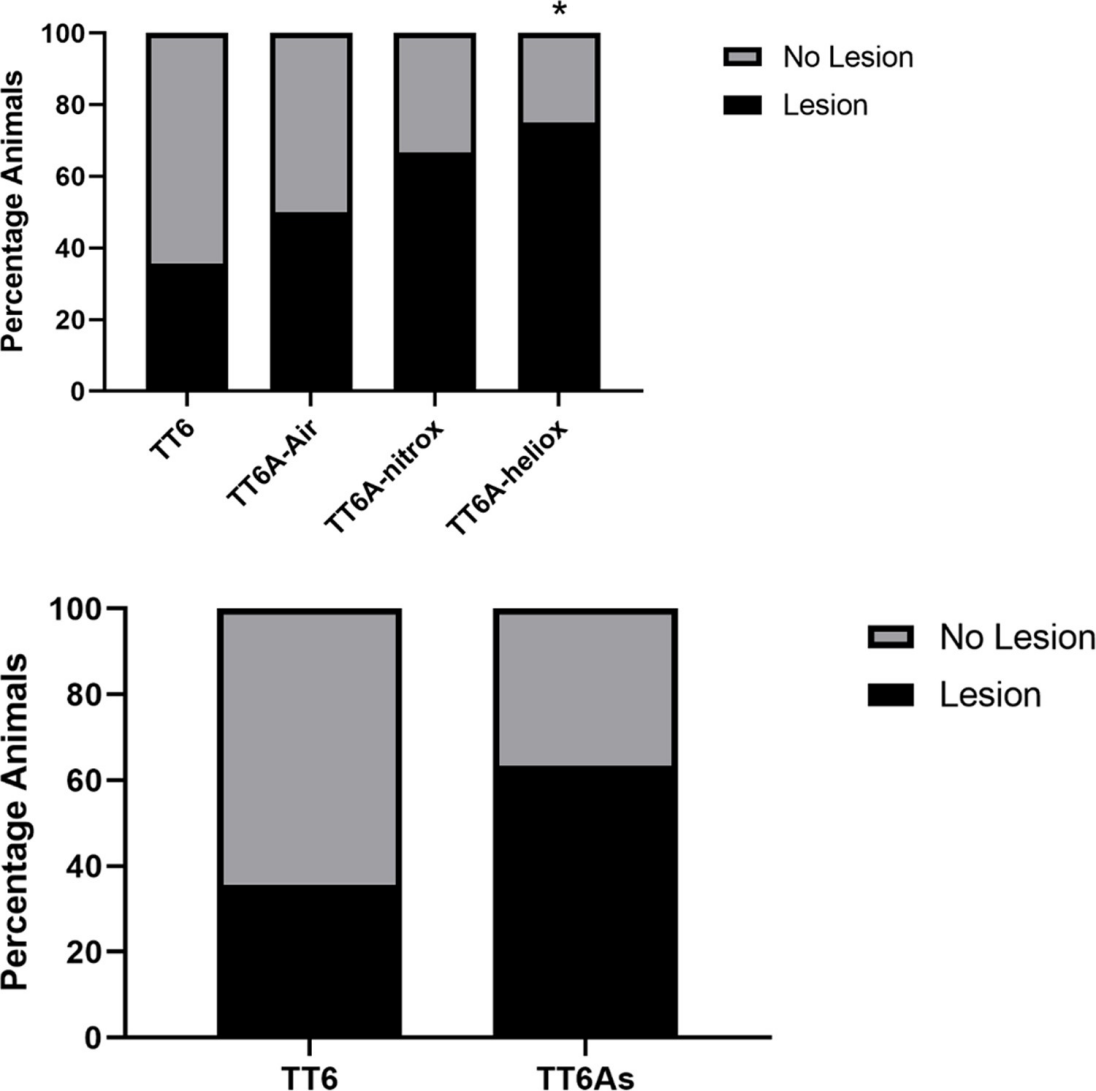
Bar graph showing the incidence of spinal cord lesion across treatment groups (A) with no significant difference among groups (chi-square test, p = 0.09), but a significant difference between TT6 and TT6A-heliox (chi-square test, p = 0.045, OR 5.4, 95% CI 0.98–29.67), and no significant difference between treatment profiles (B, Chi-Square Test, p = 0.07, OR 3.12, 95% CI 0.88–11.05).

**Fig 9:**
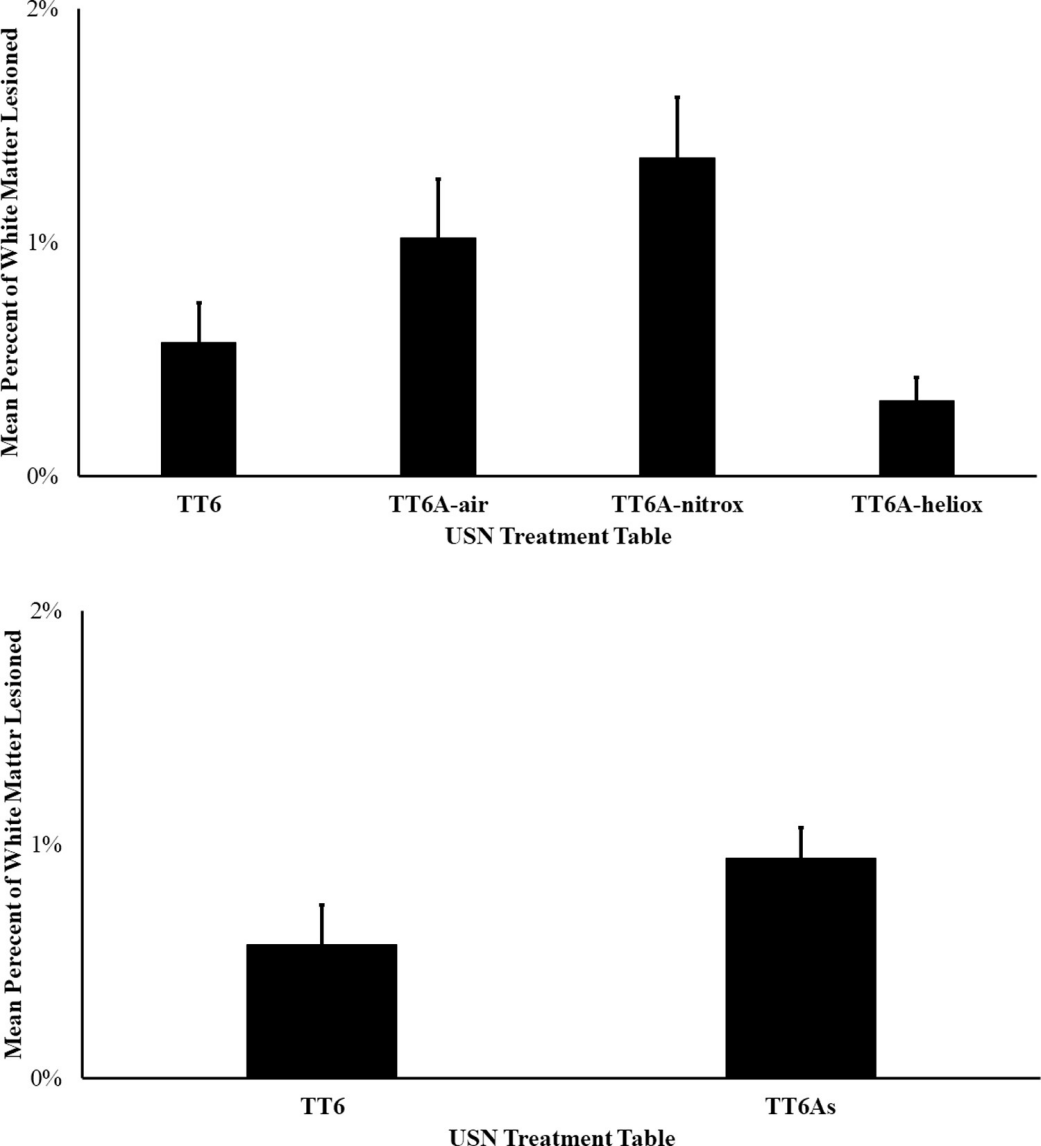
This bar graph shows the mean percent of area of white matter lesions across the different treatment groups (A, ANOVA, p = 0.51) and between treatment and between different treatment profiles (B, t-test, p = 0.17) with standard error bars and with 65% increase in mean lesion area in the TT6A profiles compared to TT6 profile.

Across all groups, we found no significant difference among lesion incidence (Chi-Square test, p = 0.17; **Fig 8A**). However, post-hoc testing identified a significant difference between the TT6 and TT6A-Heliox groups (Chi-Square test, p < 0.05, OR 5.4, 95% CI 0.98–29.67). We found no significant differences between TT6 and TT6A-air or TT6A-nitrox (Chi-Square test, p = 0.45 and 0.10, OR 1.8 & 3.6, 95% CI 0.40–8.18 & 0.78–16.67 respectively). Similarly, we found no significant difference in lesion incidence between treatment profiles (chi-squared test, p = 0.07, OR 3.12, 95% CI 0.88–11.05, **Fig 8B**). We found no significant difference among mean lesion percent area across all groups (ANOVA, p = 0.51, **Fig 9A**). Similarly, we found no difference between treatment profiles (t-test, p = 0.17, 65% increase in lesion area in TT6A profiles, **Fig 9B**). However, when we treated each spinal cord section as an individual data point, we repeated the ANOVA and found a significant difference (ANOVA, P < 0.01). A post-hoc Tukey test on this second analysis showed that TT6A-nitrox had larger lesions than TT6 and TT6A-heliox (Tukey Test, p < 0.05 with increased lesion area 139% and 325% respectively). We found no additional between group differences based on the post-hoc Tukey Test.

Finally, we found strong correlation between functional neurologic outcomes and spinal cord lesions. Specifically, we found a significant association between lesion incidence and neurologic deficit, where animals with a neurologic deficit were significantly more likely to have spinal cord lesions (Chi-Square, p < 0.01, OR 12.72, 95% CI 3.08–52.44). Similarly, we found that animals with a neurologic deficit had larger lesion areas, specifically for every one percent increase in total lesion area there was a 33% increase in odds of neurologic deficit (Logistic Regression, p < 0.0001, Model: log odds = -1.382+5.181*X, Area under the ROC curve = 0.8724, 95% confidence interval (0.7710 to 0.9737)).

## Discussion

To our knowledge, this is the first completely prospective, randomized control trial comparing both pressure and breathing gas effects through a comparison of TT6, TT6A-air, TT6A-nitrox, and TT6A-heliox after Neurological DCI caused by an air insult dive. We have demonstrated in this swine model of neurologic DCI that there is no difference in clinical outcomes after initial recompression treatment with TT6, TT6A-air, TT6A-nitrox, or TT6A-heliox. Similarly, we have found no differences in clinical outcomes between TT6 and TT6A treatment table profiles. Our study clarifies that initial management for neurologic DCI with TT6 is appropriate and raises important questions about optimizing recompression therapy for neurologic DCI. In the following discussion, we highlight the practical implications of our study findings and consider the evidence supporting theoretical benefits of recompression therapy.

### Practical implications

Neurologic DCI leads to significant mortality and morbidity in divers, yet the optimal treatment remains unknown [12,16]. There is retrospective evidence that shallower recompression may be superior to deeper recompression profiles [17]. While some hyperbaric experts prioritize pressure and others oxygen concentration gradients, neither has robust clinical evidence. A recent Cochrane Review found only one article prospectively comparing recompression therapy profiles, and the final results of this study were never published [11,12]. While the USN developed and operationalized two of the most widely used recompression treatment tables (USN TT6 and USN TT6A) in the late 1960s, the low prevalence of neurologic DCI and ethical concerns with randomizing these two treatments contribute to the lack of robust data [6]. As such, decisions regarding the use of recompression tables are based, in part, on theoretical benefits. Practically, however, our study provides strong evidence to initiate all recompression therapy for neurologic DCI with a TT6, consistent with USN Diving Manual recommendations [3]. Given the lack of differences in neurologic function among swine in the four treatment groups in our study, other factors should be considered in making the initial recompression treatment decision. The TT6 is the shortest and least resource intensive with respect to personnel, chamber specifications, and gas requirements among the four treatments we compared. Additionally, hyperbaric practitioners may infer that there may not be any benefit to recompression treatment profiles deeper than TT6 based on our data. In fact, our data suggest that deeper dive profiles may result in more spinal cord pathology. Increasing the partial pressure of the selected inert gas (e.g. nitrogen or helium) with deeper recompression profiles may contribute to additional bubble formation, increasing the risk for spinal cord pathology [18].

### Relative benefits of recompression therapy variables

Recompression treatment in part facilitates inert gas elimination to treat neurologic DCI using hyperbaric pressure and breathing gases. This treatment is targeted at reducing the size and number of bubbles in tissue or vasculature prior to downstream lesion formation. The hyperbaric pressure helps to reduce bubble size based on Boyles’ Law and to drive bubbles back into solution or tissue based on Henry’s Law. The various gas gradients, dependent on hyperbaric pressure and breathing gas mixtures, then facilitate inert gas elimination from the tissues and allow for gradual, staged ascent back to sea level. Theoretically, TT6 (partial pressure [pp] of O_2_ = 2.82 ATA = 285.73 kPa), TT6A-nitrox (ppO_2_ = 3.0 ATA = 303.98 kPa), and TT6A-heliox (ppO_2_ = 3.0 ATA = 303.98 kPa) optimize oxygen pressure gradients without an unacceptable risk of central nervous system (CNS) oxygen toxicity. Among these, only TT6 simultaneously optimizes the inert gas gradient (ppN_2_ = 0.0 ATA = 0.0 kPa). TT6A-air (ppN_2_ = 4.74 ATA = 480.28 kPa), TT6A-nitrox (ppN_2_ = 3.0 ATA = 303.98 kPa), and TT6A-heliox (ppHe = 3.0 ATA = 303.98 kPa) all have higher partial pressures of inert gas; notably, heliox has no nitrogen, the most common inert gas in diving. The relative impact of hyperbaric pressure and treatment breathing gases on clinical outcomes was previously unknown.

We might expect heliox breathing to be associated with better neurologic outcomes compared to nitrox and air. In the setting of a nitrox diving gas mixture, heliox mixtures as recompression treatment gases would be expected to maximize nitrogen elimination and thereby improve neurologic outcomes based on the principles of gas kinetics, concentration gradients, and equilibration. The solubility of helium may confer additional benefit as it has a lower solubility coefficient than nitrogen in lipids by a magnitude of 4 [19]. As such, helium reaches saturation more quickly in lipids than nitrogen. The spinal cord is lipid rich in composition and this composition is relatively highly conserved across species [20]. Helium should, therefore, confer a benefit as a diver’s breathing gas to reduce neurologic DCI and as a recompression therapy treatment gas because it will more rapidly displace and eliminate the inert gas a diver breathes compared to nitrogen. In practice, using heliox as a recompression therapy treatment gas has not changed clinical outcomes [11,16,21]. In our study, swine treated with TT6A-heliox had similar gross neurologic function and post-treatment vital signs compared with the other groups.

Our pathology data suggest that the aforementioned benefit of helium may be more due to the absence of nitrogen than the presence of helium. TT6A-heliox treated animals had the smallest spinal cord lesion areas, showing a significant difference with our secondary analysis compared to TT6A-nitrox treated animals. Similarly, animals treated with TT6 had a very similar mean lesion area compared with those treated with TT6A-heliox, suggesting that the benefit may be a result of the absence of nitrogen. In fact, the largest lesion areas appeared in the groups treated with nitrogen as the inert gas on the TT6A recompression profile, which is particularly concerning given that pathology had a strong correlation with functional neurologic outcomes across the whole dataset.

Helium’s potential benefit in washing out nitrogen may be offset by its rapid equilibration in tissues. By more rapidly reaching saturation, helium may simultaneously reduce the risk of DCI from the inert gas breathed during the dive and increase the risk for DCI from helium as the inert gas breathed during recompression therapy, especially in tissue compartments with slow gas elimination rates. Our pathology data show that TT6A-heliox treated swine had smaller spinal cord lesions but, seemingly paradoxically, had a higher incidence of spinal cord lesions. Animals treated with TT6 had significantly lower incidence of spinal cord lesions than TT6A-heliox. Helium’s low lipid solubility may explain these seemingly paradoxical results. The counter-diffusion phenomenon has been proposed as another mechanism for potentially detrimental effects of heliox recompression treatment by causing growth of nitrogen-containing bubbles. Experimental studies have yielded heterogeneous results [22–24].

The relative performances of TT6 and TT6A profiles suggest that minimizing inert gas exposure during recompression may be more important than increases in hyperbaric pressure. The USN Diving Manual recommends avoiding 100% oxygen above 2.82 ATA (i.e. maximum depth of TT6, 285.74 kPa) to avoid CNS oxygen toxicity [3]. Any recompression profile deeper than a TT6 would require the use of an inert gas mixture. The risks of additional inert gas exposure may outweigh the theoretical benefits of increased hyperbaric exposure and, as such, the therapeutic benefits of increasing pressure may have diminishing returns. Even in our study, where the maximum depth of the insult dive was much greater than 2.82 ATA (285.74 kPa), swine treated with TT6 had the same clinical outcomes and equivalent or better pathologic outcomes. While robust human data are lacking, no differences in clinical outcomes have been shown in any studies comparing TT6 to other, typically deeper recompression treatment profiles [10,16–18].

Our study did not show any differences in clinical outcomes among the different treatment groups or treatment profiles, but our pathology data provides useful evidence to support recompression therapy decisions. Swine treated with TT6 consistently had better pathologic outcomes indicating that this profile was most efficacious at reducing lesions after the insult dive. Moreover, our pathology findings strongly correlated with gross neurologic outcomes, suggesting that with more power (more swine), our study may have been able to detect a statistical significance in clinical outcomes. Consistent with the strong correlation between pathologic and clinical outcomes, swine treated with TT6 had the lowest incidence of neurologic deficit among the four treatment groups. These data provide additional evidence that providers could reasonably choose to avoid TT6A treatment profiles in favor of TT6, recognizing that while there are no data to support a clinical benefit, the pathologic data suggest that TT6A profiles may do more harm than good, at least when used as the initial recompression treatment.

### Study limitations

Animal studies provide an efficient means of providing evidence to inform clinical decision-making for the treatment of neurologic DCI. While swine are not humans, we selected a previously developed swine model for neurologic DCI with validity evidence suggesting that the model produces consistent results that align with similar human work [4,14]. We executed the model successfully as similar rates of neurologic DCI were demonstrated: 74% in the current study and 73% in the original study [4]. Our presentations were also similar with respect to severity, as evidenced from our 20% death rate and the historical death rate of 16% [4]. In this study, we excluded animals that did not complete recompression treatment. We wanted to focus on the efficacy of the recompression treatment tables for neurologic DCI and, therefore, chose to follow a per protocol analysis. The swine with the most severe manifestations of neurologic DCI encountered concomitant cardiopulmonary DCS; as a result, these swine were mostly excluded from our study, dying prior to or during recompression treatment. This aligns with our intent to focus on neurologic DCI and compromises the generalizability of our results to the most severe forms of neurologic DCI that have simultaneous presentation with cardiopulmonary DCS. Additionally, in consultation with our veterinary colleagues, we excluded three animals that had extreme outcomes (e.g., severe hyperthermia, respiratory distress secondary to pneumonia). The outcome of these animals was felt to be related to unidentified underlying pathology, rather than neurologic DCI.

Animal models, like ours, aimed at informing human clinical care inherently have limitations. The recompression table chosen were optimized for use in humans (70kg) which are much larger than 20kg swine which limits the generalizability of these findings somewhat. That said, based on prior work by Lillo et al. [25], none of the exposures in the current study would have placed the swine in a state of tissue saturation. It is likely that any differences between the 20kg swine and 70kg humans in inert gas perfusion dynamics during recompression would have been modest and preserved across treatment groups. Due to our inability to effectively assess swine for subtle deficits, we likely missed sensory changes that led to underestimated rates of neurologic DCI. While spinal cord pathology may have accounted for some of these missed sensory changes, only the animals that met our observable, motor-dominated, diagnostic criteria were enrolled and evaluated with necropsy. This means that our pathology findings account for swine that had motor deficits, with or without sensory deficits of neurologic DCI, but not necessarily swine that had sensory findings alone.

We kept the swine under sedation during recompression treatments, which is not standard of care for human recompression therapy. The length of the treatment, comfort of the swine, and experimental assets contributed to this decision. The anesthesia may have decreased swine respiratory rate and heart rate, potentially decreasing perfusion. This may have contributed to slightly higher rates of treatment failure (i.e. spinal cord pathology or gross neurologic outcomes) due to decreased perfusion and, therefore, decreased rate of inert gas elimination. To reduce impact on data, anesthesia was used consistent across all animals and treatments, and we do not believe this negatively impacted the comparison of our results.

Lastly, we enrolled fewer swine than expected and had greater variation in spinal cord pathology than expected. The slightly higher mortality rate combined with unexpected, intermittent unpredictable equipment malfunction contributed to our lower than anticipated enrollment. Additionally, we had greater variance and lower means among spinal cord lesion areas than expect based on previous studies [14]. Together, our lower enrollment, lower means, and increased variance increase the chance for a type II error, where we falsely accept that there is no difference among treatment groups or between treatment profiles. Repeating a power analysis with our results, however, suggests that we would have needed approximately 200 animals per group to identify a difference between a TT6 and each of the other groups based on lesion incidence with a power of 0.80 and alpha 0.05. Additionally, we would have needed over 600 animals per group to identify a difference between a TT6 and each of the other groups based on mean lesion area with a power of 0.80 and alpha 0.05. Importantly, even if we had reached our goal of 21 animals per group, we would not expect the results of our study to change based on our current findings. The power analysis highlights the marginal differences in outcomes among these treatment tables, supporting the utilization of the least resource intensive treatment table–TT6. That said, a clinically meaningful difference in the post treatment outcome cannot be excluded. Furthermore, future advances in lesion quantification may reduce sample sizes required to differentiate among groups.

## Conclusions

TT6 is the most appropriate initial treatment for neurologic DCI in swine, among the tables that we compared. TT6 performed as well as the other treatment tables and is the least resource intensive. While we found no difference in the functional neurologic outcomes among swine treated with TT6, TT6A-air, TT6A-nitrox, or TT6A-heliox, the pathologic data shows that swine treated with TT6 have the lowest incidence of spinal cord lesion. Moreover, the pathology data correlate with the functional neurologic outcome data, where TT6 also had the lowest rate of neurologic deficits. The combination of these pathologic and neurologic outcome data suggests that optimizing gas gradients, and specifically minimizing inert gas exposure, may be more important for improving recompression therapy outcomes than increasing pressure exposure. While not the focus of our study, our data provide limited evidence that increasing the depth of recompression therapy while breathing inert gas may worsen outcomes. For the military, our study provides preliminary evidence to support exclusive use of TT6 for diving operations, which would reduce man-hours for initial recompression treatment and technical requirements for diving recompression chambers. Future work should explore the impact of treatment table extensions, iterative recompression treatments, and intra-treatment changes in recompression dive profiles.

## Supporting information

S1 Raw data (XLSX)
